# Adherence to Positive Airway Pressure Therapy in Patients With Obstructive Sleep Apnea

**DOI:** 10.7759/cureus.25946

**Published:** 2022-06-14

**Authors:** Rabia Shaukat, Yasser Gamal, Ahlam Ali, Sherif Mohamed

**Affiliations:** 1 Department of Family and Community Medicine, UTHealth Science Center, Houston, USA; 2 Department of Pediatrics, Faculty of Medicine, Assiut University, Assiut, EGY; 3 Department of Pediatrics, Assiut University Hospital, Assiut, EGY; 4 Department of Chest Diseases and Tuberculosis, Faculty of Medicine, Assiut University, Assiut, EGY

**Keywords:** obstructive sleep apnea, bipap, continuous positive airway pressure (cpap), quality of life (qol), patient adherence

## Abstract

Adherence to either continuous positive airway pressure (CPAP) or bilevel positive airway pressure (BiPAP) therapy in patients with obstructive sleep apnea syndrome (OSAS) represents a real challenge to sleep medicine physicians. Many risk factors/predictors for nonadherence exist, and usually, it is multifactorial. Long-term nonadherence with CPAP therapy has been associated with the use of CPAP for <4 hours/night during early treatment, moderate to severe obstructive sleep apnea (OSA), poor self-efficacy, and unsupportive bed partner. The American Academy of Sleep Medicine (AASM) recommends follow-up of patients with OSA within the first two weeks of CPAP use to optimize adherence. Measures to improve adherence to positive airway pressure (PAP) therapy go through an integrated approach that involves behavioral therapy and prompt management of side effects. Pharmacologic therapy in the form of a sedative-hypnotic sleep aid has a minor role in managing nonadherence to CPAP based on the greater risk of side effects. This article will briefly discuss the risk factors and management of nonadherence to PAP therapy in patients with OSAS.

## Introduction and background

Adherence to either continuous positive airway pressure (CPAP) or bilevel positive airway pressure (BiPAP) therapy in patients with obstructive sleep apnea syndrome (OSAS) represents a real challenge to sleep medicine physicians. The most common type of sleep-disordered breathing (SDB) is obstructive sleep apnea (OSA), which implies the cessation of airflow in the presence of respiratory effort. Recurrent episodes of upper airway (UA) collapse during sleep is the characteristic feature of OSA. Recurrent desaturations and arousals from rest accompany these episodes [1].

OSA is common among older males, but it can also affect females and children. Several clinical risk factors are associated with OSA and include older age, male sex, obesity, craniofacial and upper airway abnormalities, and increased neck circumference [2]. Basic concepts in OSA treatment include elimination of contributing factors and provision of nasal CPAP (n-CPAP). This article will discuss the nonadherence of patients with OSAS to CPAP therapy, including prevalence, risk factors, and ways of management of this problem.

## Review

Nasal CPAP therapy

Historically, nasal CPAP (n-CPAP) was described in the ‘80s as the standard of care for patients with OSAS. Physiologically, CPAP, as the name denotes, implies maintaining a positive transmural pharyngeal pressure so that the intraluminal pressure exceeds the surrounding pressure. Moreover, CPAP stabilizes the upper airway via increased end-expiratory lung volume as well. Thus, respiratory events attributable to upper airway collapse/obstruction (apneas and hypopneas) can be prevented [3].

Effectiveness (Benefits) of n-CPAP

Adequate levels of n-CPAP during sleep are capable of resolving obstructive apnea and hypopnea, improving oxygen desaturation, snoring, and proper sleep continuity. Reports have observed that in OSA patients with mild and moderate apnea, the use of CPAP had improved daytime sleepiness, mood, and cognitive function [4]. CPAP has beneficial cardiovascular effects. It has also been shown to control blood pressure, primarily in patients with severe OSA. It may improve the left ventricular ejection fraction in patients with congestive heart failure and OSA and enhance right-side heart function and pulmonary hypertension [5]. CPAP has also been shown to improve quality of life. Importantly, prospective studies have observed that CPAP reduces mortality in OSA [6].

Adherence to CPAP therapy

Definition

In patients with OSA, nonadherence is defined as using CPAP for <4 hours/night or <70% of nights (i.e., <5 nights/week).

Prevalence

Unfortunately, it has been estimated that almost half of patients are nonadherent, but there is a broad range (29%-83%) [7].

How and When?

Despite its apparent assessment as a behavioral phenomenon, adherence to CPAP therapy can be objectively measured. With the mask-on time used to measure compliance, most modern CPAP devices measure both “machine-on” and “mask-on” times [5]. Adherence to CPAP may be determined as early as two weeks after CPAP is initiated, and it can predict long-term nonadherence [8].

Causes of Nonadherence

There is no single factor behind nonadherence to CPAP therapy, and it is usually multifactorial.

Risk factors/predictors: Several observational studies [7,9-20] have identified risk factors/predictors for nonadherence (Table 1).

**Table 1:** Risk factors/predictors for nonadherence to n-CPAP therapy n-CPAP: nasal continuous positive airway pressure; OSA: obstructive sleep apnea; AHI: apnea-hypopnea index

Patient-related factors
Failure to understand the importance of the therapy/instructions concerning the therapy
Moderate to severe OSA
Lower socioeconomic status
Poor self-efficacy
Social isolation
Lack of bed partner engagement
Younger age
Less severe oxyhemoglobin desaturation during sleep
Therapy-related factors
Complexity of therapy (use of device and medication dosing)
Increased rate of adverse effects
Less than four hours of CPAP nightly use in the first two weeks
Characteristics of illness (long-term or chronic illnesses)
Lack of efficacy (higher residual AHI)
Expensive therapy
Healthcare-/physician-related factors
Poor relationship/communication with the patient
Lack of knowledge of medications the patient is taking or has access to (sedatives and alcohol)
Unwillingness to educate patients

Higher residual apnea-hypopnea index (AHI) (poor efficacy of CPAP delivery) and poor self-efficacy (which involves volition, motivation, and confidence to engage in a healthy behavior) were the significant predictors of “early” nonadherence (during the first two weeks) [9,10,13]. On the other hand, long-term nonadherence with CPAP therapy has been associated with the use of CPAP for <4 hours/night during early treatment [7], moderate to severe OSA [11,12,15], lack of a passionate perspective about the benefit of CPAP therapy [16,17], poor self-efficacy [7,15-17], and unsupportive bed partner [15].

There is uncertainty about whether the routine application of heated humidification predicts adherence since the evidence is conflicting [21]. Another exciting area is the impact of different methods of CPAP delivery on commitment.

Although auto-titrating CPAP or pressure-relief CPAP is commonly prescribed in patients when nonadherence is due to intolerance of positive pressures, routine prescription of these measures was not associated with increased adherence rates compared to conventional CPAP [7,22].

Evaluation (Workup) for Nonadherence

To optimize adherence, the American Academy of Sleep Medicine (AASM) recommends follow-up of patients with OSA within the first two weeks, then monthly and yearly, depending on the level of adherence and resolution of symptoms [23]. This evaluation should focus on determining whether or not the patient is tolerating CPAP therapy, the level of commitment, perceived benefits, and identifying and troubleshooting any side effects.

Clinical assessment of patients on CPAP therapy includes interviews with the patients and their bed partner, asking about their estimated hours of nightly use, frequency of weekly service, and reasons for nonadherence, if present. The interviewer typically assesses the patient for side effects, the patient’s perception of CPAP efficacy, and the impact of CPAP on their bed partner.

Objectively, the downloaded data from the patient’s CPAP device are assessed as a measure of adherence. Newer electronic resources (telehealth, telemonitoring, and active patient engagement) may benefit the evaluation and management of compliance, particularly in the era of COVID-19 [24].

Sequalae

Subtherapeutic CPAP or “CPAP withdrawal” led to the recurrence of abnormal respiratory events within one night. Increased blood pressure, morning heart rate, and subjective daytime sleepiness were seen within two weeks of withdrawal. Psychomotor performance remained unaffected [1,4].

Management

In patients with OSA, the first-line intervention includes an integrated approach to management [7,16, 17,23,25,26] that involves behavioral therapy and prompt management of side effects, which has been shown to increase CPAP use by about one hour, sometimes more [25,26].

The American Academy of Sleep Medicine (AASM) recommends that all patients with OSA treated with CPAP receive behavioral therapy before and during the first few weeks of treatment [23]. These therapies imply cognitive-behavioral therapy (CBT) and motivational enhancement therapy (MET). CBT is a structured psychotherapeutic method used to alter attitudes and behaviors. The positive effect of CBT plus education on adherence with CPAP is likely due to improved self-efficacy (defined as one’s volition, motivation, and confidence to engage in a healthy behavior).

CBT may take many forms, including videos demonstrating CPAP use, electronic or pamphlet information, relaxation techniques, mask fitting sessions, and telephone or telehealth support. In a meta-analysis that included 41 small randomized trials of CPAP-naïve patients with OSA, behavioral therapies were associated with increased CPAP adherence (mean improvement of 50 minutes per night) [25]. The efficacy of CBT plus education has been reported in several randomized trials [26].

MET addresses the components of CPAP adherence that can be modified, such as perception and behavior. Motivational interviewing includes interaction with the patient in person and by phone during the first month to resolve perceptions, behaviors, and volitions that are barriers to engaging in the treatment. Several randomized studies have demonstrated increased CPAP use with motivational interviewing, but the effect may be lost once stopped [27].

A multidisciplinary approach to managing side effects related to CPAP therapy has proven effective in improving compliance with that therapy [7,11,12]. Interface (mask)-related problems include skin abrasions, rash, and conjunctivitis. A chin strap is advised to keep the mouth closed if there are excessive air leaks through the mouth, or an oronasal mask can be tried. In case of a poorly fitting show, patients should try masks of different sizes and models. Many patients report claustrophobia; they express that the sensation of covering the nose with an act makes them so uncomfortable to the degree that they cannot tolerate wearing the n-CPAP device [18]. Instead of a traditional nasal mask, nasal pillows may allow such patients to accept n-CPAP [18,19]. In some cases, the patient feels a sensation of increased resistance to expiration. In these cases, using a CPAP unit with a ramp feature is justifiable. This unit allows the patient to fall asleep with little or no applied pressure; then, the pressure is gradually increased to the set optimal level over a predetermined interval (which is usually 15-30 minutes). If this maneuverer did not help, then BiPAP may be used as an alternative [14,15].

Patients may experience nasal obstruction. This could be due to predominantly a fixed skeletal obstruction or a soft tissue obstruction; both are potentially modifiable without surgery. Mucosal edema is usually due to allergic rhinosinusitis or vasomotor or irritative rhinitis. Pharmacotherapy is usually enough. Patients may experience nasal dryness. This can take place where forced dry air can irritate the nose and encourage mucosal inflammation and crusting. Humidified air for n-CPAP usually eliminates this problem [21].

Significantly, active patient education and early reinforcing follow-up through behavioral therapy may improve CPAP’s long-term use and compliance [8,16,19,20].

The measures discussed above should improve patient adherence. However, 82 articles examining interventions for adherence reported that the overall CPAP nonadherence rate based on a seven hours/night sleep time was stable over 20 years at 34% [28], indicating the individualized nature of the response. Continued education and behavioral therapy are advisable for those who fail first-line interventions. Pharmacologic therapy in the form of a sedative-hypnotic sleep aid should be a last resort based on the greater risk of side effects and conflicting data regarding such therapy [29].

Notably, for patients who are adherent and in whom CPAP is adequate but remain sleepy, we evaluate for other etiologies of excessive sleepiness and suitability for a wakefulness-promoting agent.

Lastly, after apparently adequate treatment with CPAP, residual excessive daytime sleepiness (EDS) is commonly encountered. Many of these patients may not have been “optimally” treated; instead, they were given a satisfactory positive pressure to correct the respiratory events of OSA during the sleep study [1,3].

BiPAP therapy

Although n-CPAP therapy is the standard of care for patients with OSAS, some patients still require the use of BiPAP. Physiologically, in contrast to CPAP, which delivers a constant pressure during both inspiration and expiration, BiPAP allows independent adjustment of the pressures delivered differently during inspiration and expiration. These levels are set so that the expiratory positive airway pressure is directed to eliminate apneas while the inspiratory positive airway pressure is implied to eliminate hypopneas [11,12].

This feature of adjusting inspiratory and expiratory pressures independently leads to optimally lower the mean airway pressures than those of CPAP. Notably, the expiratory positive airway pressure level that must be applied is characteristically lower than the corresponding CPAP level required to maintain the patency of the airways.

BiPAP is generally indicated in patients who cannot tolerate high pressures of CPAP (i.e., those who experience difficult exhalations) or have barotrauma-related complications. Many sleep agencies automatically place a patient on BiPAP if the CPAP level needs to be increased above a certain level (15 cm water). However, it is to be realized that BiPAP is too expensive to be used as first-line therapy, yet it has no distinct advantages over CPAP therapy [11,12,30].

Importantly, it has been observed that compliance with BiPAP was not better than that with CPAP [30].

## Conclusions

Adherence to PAP therapy is of vital importance to patients with OSA. Nonadherence is usually multifactorial. The treating physician should address risk factors for such nonadherence early in using CPAP therapy, typically within the first two weeks of use. Interventions to improve nonadherence to CPAP therapy are many, among which cognitive-behavioral treatment is the most important; most of these interventions are better to be carried out through a multidisciplinary approach. Long-term monitoring is essential. Compliance with BiPAP has not been demonstrated to be better than compliance with CPAP.
